# A qualitative evaluation of barriers and facilitators to a large-scale antithrombotic stewardship intervention in the United States Veterans Healthcare system

**DOI:** 10.1007/s11096-025-01922-2

**Published:** 2025-06-04

**Authors:** Jacob E. Kurlander, Claire H. Robinson, David Parra, Lacey Evans, Von Moore, Geoffrey D. Barnes, Allison A. Ranusch, Jeremy B. Sussman

**Affiliations:** 1 VA Ann Arbor Healthcare System Center for Clinical Management Research, PO Box 130170, Ann Arbor, MI 48113-0170 USA; 2 Department of Internal Medicine, University of Michigan, Ann Arbor, MI USA; 3 Department of Veterans Affairs, Pharmacy Benefits Management, Veterans Integrated Service Network 8, Tampa, FL USA; 4 Pharmacy Benefits Management Services, VA Center for Medication Safety, Hines, IL USA

**Keywords:** Anticoagulants, Antiplatelet, Clinical pharmacists, Deprescribing, Implementation

## Abstract

**Background:**

The combined use of antiplatelet medications with direct oral anticoagulants increases patients’ risk of hemorrhage. In 2021, a multistate network of Veterans Affairs medical centers in the United States deployed a successful multicomponent stewardship initiative to reduce inappropriate anticoagulant-antiplatelet therapy.

**Aim:**

Identify barriers, facilitators, and potential adaptations of the initiative to guide broader dissemination.

**Methods:**

Clinical pharmacists and pharmacist managers were invited to participate in semi-structured interviews about their experiences with the initiative. Interviews were transcribed. Thematic analysis was informed by the Consolidated Framework for Implementation Research (CFIR).

**Results:**

Fifteen interviews were completed (response rate 68%). The initiative was considered important and worth disseminating, and the addition of a visual alert to flag potential deprescribing candidates on an anticoagulation population management dashboard was considered especially beneficial. Three primary themes were identified using the CFIR. First, pharmacists often encountered barriers to coordination across specialties, with some clinicians unconvinced of the clinical evidence favoring antiplatelet deprescribing; breaking down these clinical silos, through targeted education and identifying meaningful clinician champions, is essential to increasing success of this initiative. Second, pharmacists sought clarification about how their deprescribing efforts, a relatively new activity, would be accounted for in performance evaluations, and how clinics would meet increased staffing needs. Third, adaptability of the initiative to local context was considered valuable.

**Conclusion:**

As part of a multicomponent oral anticoagulant-antiplatelet stewardship initiative, preventing clinical silos, clarifying expectations around performance and staffing, and permitting adaptations to local context are important to maximizing impact.

**Supplementary Information:**

The online version contains supplementary material available at 10.1007/s11096-025-01922-2.

## Impact statements

- To be successful, antithrombotic stewardship interventions that involve antiplatelet deprescribing require coordination with and acceptance from relevant specialties, particularly cardiologists.
- Pharmacists tasked with leading antithrombotic stewardship initiatives must have clear performance measures and time set aside for these efforts.
- In the context of a multisite antithrombotic stewardship initiative, sites should be able to adapt the implementation strategies based on local contextual factors.

## Introduction

There is growing recognition that many patients who are prescribed anticoagulant-antiplatelet combination therapy may not have an indication for the antiplatelet medication [1–4]. In Europe, two observational studies found more than 90% of such patients used antiplatelet medications without an indication [1, 4]. Combination antithrombotic therapy is associated with higher rates of bleeding, but similar rates of thrombosis compared with anticoagulation monotherapy in patients with atrial fibrillation or a history of venous thromboembolism [2, 3, 5]. Aspirin nearly doubles the risk of upper gastrointestinal bleeding [6, 7]. Accordingly, recent European and United States (US) guidelines have narrowed the indications for combination antithrombotic therapy, and it is now generally recommended only for a limited time after vascular interventions or for vascular protection with very low dose rivaroxaban [8, 9].

It remains unknown how best to develop and scale quality improvement programs that address potentially inappropriate combination antithrombotic therapy. Deprescribing faces numerous barriers [10–12], including poor communication between clinical services, patient resistance, and clinician apprehension. Efforts to reduce inappropriate combination antithrombotic therapy carry unique challenges, including fear of cardiovascular events and ambiguity about which clinician “owns” antiplatelet management [13, 14]. For patients on combination antithrombotic therapy, one potential advantage is the existence of local structures for antithrombotic stewardship, typically anticoagulation management services led by clinical pharmacists [15]. However, the key components and contextual considerations of a pharmacist-led antithrombotic deprescribing initiative are unknown [16].

The Veterans Health Administration (VHA) is the largest integrated health system in the US and includes pharmacy services, with facility-level anticoagulation clinics focused on anticoagulation prescribing. VHA has been at the forefront of systematic antithrombotic stewardship efforts to increase the safe and effective use of anticoagulants [17–19]. The anticoagulation clinics are staffed by clinical pharmacists with expertise in anticoagulation and thrombosis management. With the ability to prescribe, they have responsibility for approving initial requests for direct oral anticoagulants (DOACs) and ensuring safe ongoing use. These clinics are led by a pharmacist anticoagulation program manager (APM) and overseen by pharmacy supervisors, who ensure comprehensive, collaborative, and patient-centered care. All VHA pharmacists have access to a DOAC population management tool, which includes an interactive electronic dashboard with a visual flag to identify potential safety issues, for example inappropriate dosing [17].

In January 2021, the anticoagulation workgroup in one regional network of VHA medical centers—Veterans Integrated Service Network [VISN] 8, encompassing seven medical centers across Florida, Georgia, Puerto Rico, and the Virgin Islands—developed an oral anticoagulant-antiplatelet stewardship initiative by conducting virtual roundtables with APMs and engaged clinical pharmacists. The overarching goal was to identify strategies to reduce antiplatelet use when an indication was lacking among patients prescribed oral anticoagulants. The final initiative included eight elements and was led by facility APMs, who could decide which elements to implement locally. The initiative components, defined in detail in Table 1, included (1) revisions to anticoagulation consults and prior authorization requests, (2) templated note language for use by the anticoagulation pharmacist in the electronic health record (EHR), (3) continuing education programs and materials, (4) identification of a clinical champion, (5) a flag added to the DOAC population management tool to efficiently identify patients using combination therapy, (6) an electronic consult to solicit cardiology input, (7) an electronic clinical reminder to primary care providers (PCPs), and (8) patient and provider correspondence templates. Challenges, opportunities, and progress were reviewed at monthly workgroup meetings, using a Plan, Do, Study, Act model of quality improvement [20].

**Table 1:** Oral anticoagulant-antiplatelet stewardship initiative elements

Name	Description
Revised anticoagulation consult and/or prior authorization requests	Verbiage added to consult and/or prior authorization request that must be completed by clinicians when first ordering a DOAC. The new verbiage asks the clinician to comment on the appropriateness of antiplatelet medications when combined with DOACs
Templated anticoagulation note language	Templated text for use in pharmacists’ notes that prompts them to assess the appropriateness of antiplatelet medications when combined with DOACs
Interprofessional education	Scheduled educational sessions to enhance the knowledge of clinical teams, often meeting criteria for continuing medical education. These sessions were often referred to as “in-services.” Information was also shared informally with clinical teams (e.g., email)
Identifying a clinical champion	Identifying and partnering with an individual outside the pharmacy service to promote and re-enforce the initiative
Antiplatelet flag	An electronic flag was added to the DOAC population management tool (dashboard) widely used by anticoagulation pharmacists throughout the VA health system. The flag identified any patient with combination therapy. The flag could be resolved by discontinuing the antiplatelet medication or be temporarily dismissed for a pre-determined duration (e.g., if an appropriate indication existed or while gathering pertinent information from a provider)
E-consult to cardiology	A method by which pharmacists or other clinicians could solicit the opinion of a cardiologist on the appropriateness of combination therapy. Cardiologists could complete e-consults through review of the medical record only, without face-to-face contact or communication with a patient
Clinical reminder^a^	A reminder directed to primary care providers in the electronic health record that prompts re-assessment of the risks/benefits of continued combination therapy
Templated letters for patients and external providers	Templated language for use in letters to patients and clinicians outside of the VA health system who may co-manage their care. The language prompted them to re-consider the appropriateness of combination therapy

*DOAC* Direct oral anticoagulant, *VA* Veteran Affairs

^a^Only assessed in the first three interviews. Subsequently, the interview guide was streamlined and omitted this question

### Aim

This qualitative evaluation sought to (1) discover the barriers and facilitators to a pharmacy-based oral anticoagulant-antiplatelet stewardship initiative within a regional network of US VHA medical centers and (2) describe potential adaptations likely to maximize success.

### Ethics approval

This program evaluation qualified as a non-research quality improvement activity conducted under the authority of VHA operations per the VHA Program Guide 1200.21, as the purpose of the project was to support internal evaluation efforts and develop recommendations for wider VHA dissemination. Thus, Institutional Review Board review was not required.

## Method

### Sampling

This work is part of a concurrent mixed methods study of the oral anticoagulant-antiplatelet stewardship initiative in Veterans Integrated Service Network (VISN) 8. Purposive sampling techniques were used to recruit one APM, pharmacy supervisor, and anticoagulation clinical pharmacist from each site to explore their detailed perspectives based on direct involvement with the initiative. Recruitment outreach included up to three emails containing a project description and the interview guide attached for potential interviewees’ review.

### Data collection and analysis

Operational partners helped develop the interview guide, which focused on identifying barriers and facilitators to successful implementation of the initiative (Supplementary File 1). After completing three interviews, the interview guide was further streamlined with input from the operational partners. From March–May 2023, CHR and LE conducted interviews using Microsoft Teams (Supplementary File 2). Verbal informed consent was obtained from all interviewees and audio-recorded at the start of each interview. Interviews were audio-recorded and transcribed verbatim. Next, JEK, CHR, LE, and AR independently performed thematic coding of three transcripts in Microsoft Word using constructs from the updated Consolidated Framework for Implementation Research (CFIR) [21, 22] and the initiative components as the coding framework (Supplementary File 3). The updated CFIR is a framework designed to systematically identify potential implementation barriers and facilitators [22], which might inform necessary adaptations prior to wider dissemination. Meetings were held to establish coding consensus, in which codes were refined and new codes were generated as necessary. All remaining transcripts were coded in dyads comprised of CHR, LE, and AR, before being uploaded to NVivo qualitative data analysis software. CHR, LE, and AR then used a consensus approach using two code reports to create and refine a template to facilitate code summaries, individually summarized the remaining code reports (all eight initiative components and eleven of the most salient CFIR constructs), and regularly met to discuss interpretation and iteratively update the template and final themes.

## Results

Of 22 individuals recruited, 15 semi-structured virtual interviews were conducted with eight APMs, two pharmacy supervisors, and five anticoagulation clinical pharmacists from the seven VISN 8 medical centers to identify barriers and facilitators to use of the initiative elements, and opportunities for improvement; participation and participant characteristics are shown in Table 2. The response rate was 68.2% and interviews lasted an average of 54 minutes (range 35–63 minutes).

**Table 2:** Participants

Site	A	B	C	D	E	F	G	Total
Number of interviewees	3	3	1	2	3	1	2	15
Number invited	3	3	3	3	4	3	3	22
Response rate	100.0%	100.0%	33.3%	66.7%	75.0%	33.3%	66.7%	68.2%
*Interviewee roles*								
APM	1	1	1	1	2	1	1	8
Pharmacist	1	1	0	1	1	0	1	5
Supervisor	1	1	0	0	0	0	0	2

*APM* Anticoagulation program manager

Participants resoundingly felt the oral anticoagulant-antiplatelet stewardship initiative was important, kept co-prescribing of antiplatelets at the forefront of clinical decision making, and should be implemented enterprise wide. However, three primary themes, each comprising sub-themes, emerged from their reflections and experiences that point to important considerations before wider initiative dissemination: preventing clinical silos (lack of coordination between disciplines and specialties), clarifying expectations, and allowing adaptations. Elaboration on these themes and illustrative quotations are provided below. These findings were derived from our coding process using the updated CFIR and primarily map to the Inner Setting domain (Supplementary File 3).

### Preventing clinical silos

While the initiative was designed to be led by pharmacists, interviewees expressed a strong need for multi-disciplinary clinical partnerships for the initiative to be successful. Prescribers, including PCPs, cardiologists, neurologists, vascular surgeons, and hematologists, must accept the evidence-base and then collaborate with pharmacists across clinical services to evaluate patients’ need for co-prescribing DOACs and antiplatelet medications. Poor communication resulted in an inefficient and sometimes ineffective review and subsequent deprescribing process.

#### Accept the need for deprescribing

Interviewees stated that cardiologists had not universally embraced that all co-prescribing of DOACs and antiplatelet medications must be assessed for appropriateness. However, recent professional guidance helped improve cardiologist acceptance of the need to evaluate co-prescribing.

“… a lot of our resistance has been trying to get cardiology onboard with new clinical guidelines and new literature that’s coming out. […] [We had some] cardiology physicians tell us until the VA came out with guidance, they weren’t going to change their practices.” (ID R4104)

Many interviewees felt interprofessional education (also called in-service trainings) should focus not just on the initiative, but more on the underlying clinical evidence to help educate, engage, and connect all relevant clinical services.

“In-services […] on the updated societal guidance is critical […]. When we presented again on not having the need of aspirin therapy and primary prevention in certain situations, we got a ton of questions on: Well, what happened to the old studies and why are those no longer valid […]?” (ID R4104)

#### Collaboration

While anticoagulation pharmacists have scopes of practice to provide comprehensive medication management, deprescribing, when indicated, is most safely and effectively carried out as part of a care team, which can bring together complementary medical skills. In the absence of input from their colleagues, anticoagulation pharmacists often felt uncomfortable making deprescribing decisions, especially when they had no pre-existing relationship with the patient.

“…oftentimes these are decisions that the pharmacist cannot make independently and so it’s important that the providers […] that are involved in the care of the patient also step up to what they need to do in their role in making that decision, because it’s really a shared decision.” (ID R3217)

For some patients, anticoagulation pharmacists struggled to get detailed information about the clinical indication for combination therapy, making it difficult to determine the appropriateness of deprescribing. This was particularly challenging when prescriptions originated from non-VA providers and were not adequately documented in the VA EHR.

“A lot of people will document that the patient’s on aspirin but not add it to the non-VA med list, so then other providers don’t even know that the patient is on concomitant antiplatelet therapy. […] your flags are only as accurate as our documentation, so if we’re not documenting it, it’s not going to flag…” (ID R1316)

Several interviewees felt strongly that a champion for the initiative would facilitate communication between clinical services. The champion should be a local, respected, trusted, and enthusiastic physician volunteer who could garner acceptance from other physicians, foster ongoing collaboration, provide stewardship, and review difficult cases.

“Having a specialist like a hematologist or cardiologist or someone who cares about the topic would be ideal because they can bring new ideas on their side so I could bring ideas on the pharmacy side. […] I think in a perfect world, you’d have somebody who’s practicing in some capacity with patients on anticoagulants.” (ID R6108)

Pharmacists felt they did not have the reach or clout that a physician champion would and felt limited in their ability to influence prescribing behaviors.

“…we have a very divided cardiology service on the risks associated with antiplatelets and anticoagulants. […] I think from a pharmacist’s perspective, we can share literature, we can share our knowledge, but I think having a physician level speak on the risks and benefits and the current literature that’s out there, we may be able to get a more united cardiology service, and then primary care would then buy in.” (ID R1101)

### Clarifying expectations

While anticoagulation pharmacists greatly valued the addition of the antiplatelet flag (Table 3), which identified patients prescribed antiplatelet medications on the DOAC population management dashboard, many noted apprehension the day the flag was added to the dashboard. They felt that more could have been done to prepare pharmacists, prescribers, and leaders to set expectations for antiplatelet flag performance metrics, the role of performance metrics in pharmacists’ evaluations, and determining appropriate staffing. It is important to note that a performance metric was intentionally not established for the initiative as it was unknown what an appropriate rate of co-prescribing would be. However, it was clear that communication with anticoagulation pharmacists about expectations could have been improved.

**Table 3:** Oral anticoagulant-antiplatelet stewardship initiative elements in use and endorsement for broader dissemination by site

Site		A	B	C	D	E	F	G
Initiative Element	In Use; Endorsed for broader dissemination							
Revised templated text for anticoagulation consults and/or prior authorization drug requests	In use	Yes	Yes	Yes	No	Mixed	Yes	Mixed
	Endorsed	Yes	Yes	Yes	Yes	Yes	Yes	Yes
Revised templated text for anticoagulation note templates that addressed DOAC-antiplatelet use	In use	Yes	Yes	Yes	Yes	Yes	Yes	Yes
	Endorsed	Yes	Yes	Yes	Yes	Yes	Yes	Yes
Informed other services/ provided educational session(s) about initiative	In use	Yes	Yes	Yes	Yes	Yes	Yes	Yes
	Endorsed	Yes	Yes	Yes	Yes	Yes	Yes	Yes
Identified non-pharmacy champion and/or partnered with another service to support the initiative	In use	No	No	No	No	No	No	Yes
	Endorsed	Yes	Mixed	No	Mixed	Yes	Mixed	Mixed
DOAC dashboard antiplatelet flag incorporated into processes	In use	Yes	Yes	Yes	Yes	Yes	Yes	Yes
	Endorsed	Yes	Yes	Yes	Yes	Yes	N/A	Yes
e-Consult to Cardiology or other service to address combination therapy	In use	No	No	No	No	No	Yes	No
	Endorsed	No	No	Yes	N/A	Yes	Yes	No
Provider and patient letters for Care in the Community or non-VA provider (self-directed care)	In use	Mixed	No	Yes	No	No	Yes	No
	Endorsed	Mixed	No	Yes	Mixed	Yes	N/A	No

Majority indicated use/endorsement = yes

Divided indication of use/endorsement = mixed

Majority did not indicate use/endorsement = no

*N/A* Not assessed

#### Metrics

Anticoagulation pharmacists had been accustomed to being appraised on their ability to minimize the number of flags on the DOAC population management dashboard, with some perceiving an implicit goal of zero flags. However, when the antiplatelet flag was initially added to the dashboard, hundreds of flags were added at once, which created angst and the need for input from leadership on how to manage this large number of flags. Furthermore, pharmacists were often unable to resolve the flags without input from another clinician on the indication and intended duration of antiplatelet treatment, which was a source of consternation.

“[…] I don’t think we were prepared that this was going to go live, and we had been trying to previously […] to keep them [flags] as close to zero as we can, and they dumped a big number of flags on us. It was a little bit of a shock to us, but we reached out to management, and they’re like, “Don’t worry, just do them as you as you go through them. We’re not going to hold you responsible for getting all of these done right now.” (ID R4223)

#### Performance evaluations

Anticoagulation pharmacists and their supervisors noted a lack of clarity on if or how the antiplatelet flag should be addressed in annual performance evaluations. Incorporating the antiplatelet flag into performance evaluations was not suggested as part of the initiative. However, interviewees desired clearer guidance on flag clearing metrics and noted that metrics should be adjusted as workload and processes related to the flag were established.

“…there’s no clear guidance on how much needs to be done, which ones [flags] need to be done. These are things that we’ve implemented locally on how to tackle them, you know critical flags first […] all we can do is quantify the notes and that doesn’t necessarily tell us how many of these particular flags they did versus another. So, the reporting of being able to capture something like that where we can assess it and score or rate it is not feasible at this moment.” (ID R2411)

Pharmacists found addressing the antiplatelet flag often took longer than addressing other flags, such as those relating to appropriate dosing or lab monitoring. If performance evaluations credited all flags equally, this could create an incentive to deprioritize addressing the antiplatelet flag.

“If I’m not a pharmacist who’s cranking out a patient every 15 minutes and they’re behind, the last thing they’re going to want to do is grab an aspirin flag because that’s not an easy flag, and I’m saying that because I cover clinic, that’s why I like managing the clinic because I work in the clinic too, so I know what flags are not fun” (ID 3103)

#### Staffing

Several interviewees mentioned the need for additional staffing in the anticoagulation clinic to address DOAC-antiplatelet co-prescribing if the initiative were to continue as a pharmacy-based intervention.

“… if pharmacy is going to be the group that is responsible for the improvement of metrics, then there needs to be an additional role or additional assistance to make that happen versus the anti-coagulation pharmacist. The anti-coagulation clinic is a grind, like I told you, I think I’m well-staffed, but they are burned out and busy and it’s a grind and when you are busy and you get pushback from a provider, it’s very difficult to continue to go into press and you’re kind of like, “Well, I asked,...” (ID R1410)

### Allowing adaptations

The initiative elements were generally found to be useful, and interviewees considered several to be essential for wide-spread dissemination, especially the antiplatelet flag, which aided in identifying patients with potentially inappropriate combination antithrombotic therapy (Table 3). Still, most interviewees strongly felt that, in the event of broader dissemination, adoption of initiative components should remain voluntary, and facilities should have flexibility to adapt them to local workflows.

“If it’s consultative, “Hey, you know this is a way that you might address this, you might consider these different approaches,” and leaving it up to the facility to implement the approaches that they think will work best given their knowledge of the environment, I think is helpful. [….] Resources are helpful […] but requirements to utilize all resources are not always helpful.” (ID R1410)

Interviewees reported that cardiologists at several sites were hesitant to implement an electronic consult (e-consult) specific to combination therapy to allow non-cardiologists to obtain input on DOAC-antiplatelet co-prescribing because of concern for excessive workload. Instead, existing communication strategies were used.

“The cardiology team did not want a consult for that. Now, they also are normally pretty good at responding when we communicate in other ways, so I was okay with that. If the VISN required it, I would absolutely love it, but again, it’s really hard because our services have to work well together, and I just didn’t think that was worth the battle at this time. It was more of a, “If you don’t want a consult then I need you to respond to my addendums,” or “If you don’t want a consult, then when I message you in Teams, I need you to look at that,” and that has worked pretty well.” (ID R3103)

Some sites did not implement the revised anticoagulation consult language, which added verbiage asking prescribers to comment on the appropriateness of antiplatelet medications when first prescribing a DOAC, because they found it excessively long. Without it, anticoagulation pharmacists had to contact prescribers to gather this information.

“So, we don’t necessarily use the updated text in our consults itself, mainly because we got pushback from Primary Care on the consults being too cumbersome or long or too many clicks...” (ID R4104)

Some interviewees described innovations not available at the time the initiative was rolled out, including a report that allowed pharmacists to track and compare co-prescribing data relative to other facilities in the VISN.

“…it helps you look at how you’re doing in relations to your other VISN [regional] partners, and not that it’s a competitive thing, but it does put things into perspective, right? We always want to shoot for zero as it were, a hundred percent, but we know that’s not realistic, but at least you can see, well, if you’re a big outlier, like okay there’s something that’s going on here...” (ID R2102)

As part of the initiative and within their scope of practice, some sites encouraged anticoagulation pharmacists to deprescribe antiplatelet medications independently in selected circumstances, for example patients using aspirin for primary prevention of cardiovascular disease. While this could circumvent the reliance on other clinicians, most pharmacists were not comfortable deprescribing independently and preferred to make these decisions collaboratively. Since the pharmacists often did not have a pre-existing relationship with the patient, many still wanted input from a cardiologist or PCP to be certain that they were not overlooking an appropriate indication for antiplatelet therapy.

## Discussion

### Principal findings

This study explored how frontline pharmacists and pharmacy leaders perceived a multi-component VHA initiative to improve appropriate use of antiplatelet medications in patients prescribed DOACs. While pharmacists were broadly supportive of the initiative and endorsed dissemination, they also sometimes found participation in the initiative challenging and time-consuming because of limited collaboration from clinicians, who frequently questioned the evidence base and failed to engage in multi-disciplinary decision-making. Pharmacists also wanted clear deprescribing metrics along with guidance on how anticoagulation clinics, efforts should be organized and incentivized. Finally, we found that interviewees preferred the flexibility to adapt the initiative to local needs. Our findings demonstrate the value of small-scale tests of change prior to broad dissemination.

### Comparison to prior literature

Our finding of challenging coordination of care between disciplines and varying support for antiplatelet deprescribing across specialties aligns with prior studies. A qualitative study of pharmacists in Saudi Arabia found that challenges collaborating effectively with physicians contributed to potential safety issues in DOAC prescribing [23]. A cross-sectional US survey of pharmacists and clinicians, predominantly in academic settings, found high rates of disagreement about when combination therapy is indicated, even when there is no compelling aspirin indication [24]. Furthermore, pharmacists were more likely than physicians to recommend deprescribing for patients taking aspirin for primary prevention. A 2022 systematic review of patient, caregiver, and provider barriers and facilitators to deprescribing cardiovascular medicines found uncertainty about when deprescribing is appropriate, fear of negative consequences, and social influences to be the most common barriers [25].

Insufficient coordination of care has been a persistent barrier to transformation efforts, including in quality and patient safety [26]. A Canadian policy synthesis on teamwork in healthcare found that “teams work most effectively when they have a clear purpose; good communication; coordination; protocols and procedures; and effective mechanisms to resolve conflict when it arises” [27]. These elements were difficult to achieve in the oral anticoagulant-antiplatelet stewardship initiative. Leadership and commitment by clinical service lines outside of pharmacy will be essential to maximizing the success of similar initiatives [20].

Our finding that pharmacists preferred to adapt the initiative to their own sites corroborates prior literature on the importance of context to implementation success [28]. Some elements were nearly universally adopted, including interprofessional education about the initiative, revising templated language in requests for DOAC approval to address antiplatelet medications, and adding a flag to identify antiplatelet users to a DOAC population management dashboard. These should be considered the core elements for future antiplatelet safety initiatives.

While interviewees desired greater clarity about the target number of antiplatelet flags and how they would factor into performance appraisals, these questions are not straightforward. Unlike many performance indicators in health care, some patients should receive combination therapy, and the appropriate number will vary with the local patient population and practice patterns. As such, a target was neither proposed nor encouraged. Instead, pharmacists might benefit from process-related targets regarding the number of attempts to resolve the antiplatelet flags. Alternatively, the flag could be modified to indicate only patients for whom antiplatelet medications are almost certainly unnecessary (e.g., for primary prevention of cardiovascular disease), in which case near zero would be a reasonable target.

### Limitations

This study had several limitations. First, study participants were limited to pharmacy staff and leadership, and did not include physicians. While pharmacists cannot speak for other clinicians, our findings reflect the experiences and perspectives that pharmacists had in their interactions with them. Second, VHA is an integrated health system in which pharmacists are often given substantial clinical independence, similar to some other countries, like the United Kingdom, where pharmacists may prescribe autonomously within their scope of practice [29]; our findings may not be generalizable to health systems where pharmacists, prescribing powers are more circumscribed. However, pharmacists in the US and elsewhere are playing a greater role in clinical care than in the past [30]. Third, data saturation was not assessed in the 15 interviews carried out. However, this did not preclude achieving the exploratory aims of the project.

### Implications

These limitations notwithstanding, our findings have implications for enhancing future dissemination of the oral anticoagulant-antiplatelet stewardship initiative in VHA and for deprescribing programs in general. If pharmacists cannot deprescribe antithrombotic medications independently, it is essential that they share an understanding with prescribers about the evidence and indications for combination therapy. This could be achieved in several ways. Professional organizations should continue to include such recommendations in relevant guidelines. The VHA independently conducts evidence-based syntheses to inform national practice guidelines. They should highlight evidence from randomized trials, which have shown that rivaroxaban monotherapy was non-inferior to combination therapy for efficacy and superior for safety in patients with atrial fibrillation and stable coronary artery disease [5]. Since there are few randomized trials of deprescribing interventions, which are typically required for the strongest (Grade I) recommendations, high-quality indirect evidence (observational studies) should also be reviewed [2, 3]. Future deprescribing efforts would also benefit from close coordination between pharmacy, cardiology, and primary care leaders, who should emphasize the high priority of initiatives before they begin, and identifying a proactive clinical champion, ideally a cardiologist. When evidence clearly supports antiplatelet deprescribing, empowering pharmacists to do so independently might accelerate dissemination.

## Conclusion

Pharmacists, accomplishing the goals of the oral anticoagulant-antiplatelet stewardship initiative depended on robust collaboration and upfront acceptance from specialty clinicians, clear performance goals, dedicated time to participate, and the ability to adapt initiative components. These findings reinforce the importance of understanding and addressing contextual factors of the health care setting and robust clinical teams with defined roles and workflows for the success of future oral anticoagulant-antiplatelet stewardship efforts.

## Supplementary Information

Below is the link to the electronic supplementary material.

Supplementary file1 (DOCX 21 kb)

Supplementary file2 (DOCX 20 kb)

Supplementary file3 (DOCX 23 kb)
