# Supplementary material for: A qualitative evaluation of barriers and facilitators to a large-scale antithrombotic stewardship intervention in the United States Veterans Healthcare system
Source: Int J Clin Pharm. 2025 Jun 4;47(6):1710–9. doi: 10.1007/s11096-025-01922-2 (PMC12630269; doi:10.1007/s11096-025-01922-2)
Supplement: Supplementary file 1 — Supplementary file1 (DOCX 21 kb) [file 11096_2025_1922_MOESM1_ESM.docx]

**Supplementary File 1: Interview Guide**

My name is [interviewer name] and I am from the VA in Ann Arbor, Michigan. With me are [2^nd^ interviewer name] and [note taker name]. We are part of the MIDAS QUERI team made up of researchers and physicians based at the Center for Clinical Management Research at the Ann Arbor VA. MIDAS is a quality improvement program partnering with National Pharmacy Benefits Management (PBM) and the VISN 8 PBM Pharmacy Anticoagulation Workgroup to evaluate the rollout of VISN 8’s Safe Prescribing Initiative on co-prescribing of antiplatelets with direct oral anticoagulants. As part of this initiative, the VISN 8 workgroup created multiple tools available to VISN 8 medical centers to help address safe co-prescribing of these medications. We are interested in learning from you which of these tools are used at your medical center and how well they have or have not worked for you. The results of our evaluation will help National PBM and VISN 8 identify opportunities for improving the initiative and determine whether a similar initiative should be rolled out nationally.

Taking part in this interview is completely voluntary. Anonymity and confidentiality of participants will be preserved by limiting access to identifiable information to only our project team. Your participation will not be shared with your supervisors, National PBM, or VISN 8 PBM and will not affect your employment at VA. Findings shared outside of our team will be de-identified and reported in aggregate at the facility level. There are no right or wrong answers and we encourage you to provide candid responses about your experiences.

The interview will last no longer than 1 hour. You can skip any questions you prefer not to answer. You can stop the interview at any time. We would like to record this interview so that we have a complete and accurate record of the information you provide. You may ask to pause or stop recording at any time. The audio file will be stored in a restricted access file on a secure VA server with access limited to a select set of team members.

Do I have your permission to record the interview?

Do you have any questions before we get started?

[Turn on recorder] Okay, to confirm, I’m starting the recording. Is this ok with you?

1. Please tell me your title and role in VA.
   1. How long have you been in this position?
   2. How long have you worked at this facility? (Where did you work prior?)

**I’d like to start by asking you some structured questions about which elements of the initiative are in use at your facility. Then, we’ll move on to a more open discussion about the details of what is and isn’t working well and why.**

1. Please tell me which of these elements of the initiative are in use at your facility:
   1. Revised templated text for anticoagulation consults and/or prior authorization drug reviews (PADR) that addressed DOAC-antiplatelet use.
   2. Revised templated text for in/outpatient anticoagulation note templates/view alerts that addressed DOAC-antiplatelet use.
   3. Informed other services/provided an in-service about initiative
      1. Who did you inform?
      2. When?
      3. How?
      4. Distributed Anticoagulation Forum Rapid Resource?
   4. Identified a specific oral anticoagulation-antiplatelet champion and/or partnered with another service to support the initiative. (What was the clinical role of the champion?)
   5. DOAC population management tool DOAC-antiplatelet flag incorporated into processes
   6. Created an e-Consult to Cardiology or other service (specify) to address combination therapy
   7. Provider and patient letter examples for Care in the Community or non-VA provider (self-directed care)

**Now, I’d like to ask some follow up questions to better understand your experiences with the DOAC-antiplatelet initiative and how things are working at your facility and why.**

1. Please describe your facility’s approach to addressing patients that are flagged for using antiplatelet medications in addition to DOACs?
   1. How has your experience been using this process? What has the reaction been from frontline pharmacists who use the PMT?
   2. Specific strategies for addressing flags?
   3. Who else is involved in addressing the care when a flag is activated?
2. Other than the strategies we reviewed at the beginning of the interview, does your medical center have other processes to ensure that patients newly initiated on DOACs are on antiplatelet medications only if clinically indicated?
3. Which of the initiative elements is most useful for your facility’s approach to addressing patients that are flagged? Why?
   1. Least useful? Why?
   2. Which elements have the biggest effect on clinical outcomes? Why?
   3. What are your suggestions for improving the process of addressing patients that are flagged?
4. Are there specific workload challenges related to DOAC-antiplatelet management?
   1. If so, how if at all, have you addressed them? Were additional staff required?
      1. What changes would you recommend?
5. Are there specific organizational challenges related to DOAC-antiplatelet management?
   1. If so, how if at all, have you addressed them?
      1. What changes would you recommend?
6. Would you recommend that the antiplatelet flag be turned on for the DOAC PMT tool nationally?
   1. Why or why not?
   2. What changes, if any, would you recommend before the flag is turned on nationally?
7. What have I not asked about that you think is important for us to understand as we continue to evaluate the Safe Prescribing Initiative on co-prescribing of antiplatelets with direct oral anticoagulants?
8. Who else in your medical center would you recommend we talk with to learn more about how the DOAC-antiplatelet initiative worked at your facility?

**Article information:**

Barriers and facilitators associated with a large-scale antithrombotic stewardship intervention to improve appropriate use of combination anticoagulation-antiplatelet therapy: a qualitative study

*International Journal of Clinical Pharmacy*

Jacob E. Kurlander^1, 2^; Claire H. Robinson^1^; David Parra^3^; Lacey Evans^1^; Von Moore^4^; Geoffrey D. Barnes^2^; Allison A. Ranusch^1^; Jeremy B. Sussman^1,2^

1. VA Ann Arbor Healthcare System Center for Clinical Management Research
2. Department of Internal Medicine, University of Michigan, Ann Arbor, MI, USA
3. Department of Veterans Affairs, Veterans Integrated Service Network 8, Pharmacy Benefits Management, Tampa, FL
4. VA Center for Medication Safety, Pharmacy Benefits Management Services, Hines, Illinois

**Corresponding Author:**

Jacob Kurlander, MD, MS

VA CCMR, VA Ann Arbor Healthcare System (152)

PO Box 130170

Ann Arbor, MI 48113-0170

jkurland@med.umich.edu
