# Supplementary material for: A qualitative evaluation of barriers and facilitators to a large-scale antithrombotic stewardship intervention in the United States Veterans Healthcare system
Source: Int J Clin Pharm. 2025 Jun 4;47(6):1710–9. doi: 10.1007/s11096-025-01922-2 (PMC12630269; doi:10.1007/s11096-025-01922-2)
Supplement: Supplementary file 2 — Supplementary file2 (DOCX 20 kb) [file 11096_2025_1922_MOESM2_ESM.docx]

**Supplementary File 2: Consolidated Criteria for Reporting Qualitative Studies (COREQ): 32-item checklist^a^**

| **No** | **Item** | **Guide questions/Description** | **Response/Page number** |
| --- | --- | --- | --- |
| **Domain 1: Research team and reflexivity** | | | |
| Personal Characteristics | | | |
| 1. | Interviewer/facilitator | Which author/s conducted the interview or focus group? | CHR and LE conducted the interviews. |
| 2. | Credentials | What were the researcher's credentials? *E.g., PhD, MD* | CHR and LE both have a MPH. |
| 3. | Occupation | What was their occupation at the time of the study? | CHR and LE are Research Health Science Specialists. |
| 4. | Gender | Was the researcher male or female? | CHR and LE are both female. |
| 5. | Experience and training | What experience or training did the researcher have? | CHR has 27 years of experience in Health Systems Research, much of which has been focused on qualitative data collection and analysis, has a certificate in Qualitative Methods from the University of North Carolina, and is master’s level trained in public health (MPH).  LE has 6 years of experience in Health Systems Research, about half of which has focused on qualitative data collection and analysis. She is master’s level trained in public health (MPH). |
| Relationship with participants | | | |
| 6. | Relationship established | Was a relationship established prior to study commencement? | No, a relationship was not established prior to study commencement. |
| 7. | Participant knowledge of the interviewer | What did the participants know about the researcher? e.g., personal goals, reasons for doing the research | Participants knew that both CHR and LE worked for the Veterans Health Administration. |
| 8. | Interviewer characteristics | What characteristics were reported about the interviewer/facilitator? e.g., Bias, assumptions, reasons, and interests in the research topic | No characteristics were reported about CHR or LE. |
| Domain 2: Study Design | | | |
| Theoretical framework | | | |
| 9. | Methodological orientation and Theory | What methodological orientation was stated to underpin the study? e.g., grounded theory, discourse analysis, ethnography, phenomenology, content analysis | The updated Consolidated Framework for Implementation Research (CFIR) constructs were used to understand perceptions of barriers and facilitators while remaining open to new inductively informed themes. |
| Participant selection | | | |
| 10. | Sampling | How were participants selected? e.g., purposive, convenience, consecutive, snowball | Purposive sampling was used to recruit anticoagulation clinical pharmacists, program managers, and supervisors across seven medical centers within one VHA region. |
| 11. | Method of approach | How were participants approached? e.g., face-to-face, telephone, mail, email | Participants were invited to participate in an interview via e-mail. |
| 12. | Sample size | How many participants were in the study? | 15 interviewees were included in the study. |
| 13. | Non-participation | How many people refused to participate or dropped out? Reasons? | 6 people passively refused to participate by not responding to the email invitations and 1 person declined participation suggesting another colleague be interviewed instead. |
| Setting | | | |
| 14. | Setting of data collection | Where was the data collected? e*.g., home, clinic, workplace* | Data were collected virtually using Microsoft Teams with the interviewer and interviewee in their respective physical or virtual workplaces. |
| 15. | Presence of non-participants | Was anyone else present besides the participants and researchers? | MS took comprehensive notes and managed the recording during the interviews. |
| 16. | Description of sample | What are the important characteristics of the sample? *e.g., demographic data, date* | The important characteristics of the sample are sites in the region that participated in the initiative and the role of the interviewee (anticoagulation pharmacist, program manager, and supervisor). |
| Data collection | | | |
| 17. | Interview guide | Were questions, prompts, guides provided by the authors? Was it pilot tested? | The interview guide was provided to participants in advance of the interviews. It was not pilot tested but was reviewed and edited by operational partners. |
| 18. | Repeat interviews | Were repeat interviews carried out? If yes, how many? | No repeat interviews were carried out. |
| 19. | Audio/visual recording | Did the research use audio or visual recording to collect the data? | Interviews were audio recorded. |
| 20. | Field notes | Were field notes made during and/or after the interview or focus group? | MS took comprehensive notes during the interviews to serve as an interim data source while waiting for verbatim transcription. |
| 21. | Duration | What was the duration of the interviews or focus group? | Interviews lasted an average of 54 minutes, ranging in length from 35 to 63 minutes. |
| 22. | Data saturation | Was data saturation discussed? | No, data saturation wasn’t discussed; however, deep understanding of the topic was gained. |
| 23. | Transcripts returned | Were transcripts returned to participants for comment and/or correction? | Transcripts were not returned to participants. |
| Domain 3: Analysis and findings | | | |
| Data analysis | | | |
| 24. | Number of data coders | How many data coders coded the data? | CHR, LE, study PI (JEK) and one other research team member (AAR) participated in initial consensus coding. CHR, LE, and AAR coded the remaining data in dyads. |
| 25. | Description of the coding tree | Did authors provide a description of the coding tree? | The coding tree includes the updated CFIR constructs and the initiative elements. Additional descriptive codes were added inductively. The coding tree is included as Supplementary File 2. |
| 26. | Derivation of themes | Were themes identified in advance or derived from the data? | Themes were derived from the data. |
| 27. | Software | What software, if applicable, was used to manage the data? | NVivo 12 Pro was used to manage the data. |
| 28. | Participant checking | Did participants provide feedback on the findings? | No, participants did not provide feedback on the findings. |
| Reporting | | | |
| 29. | Quotations presented | Were participant quotations presented to illustrate the themes / findings? Was each quotation identified? e*.g., participant number* | Yes, participant quotations were presented to illustrate findings and were identified using a unique participant identification number. |
| 30. | Data and findings consistent | Was there consistency between the data presented and the findings? | Yes, the research team met to ensure there was consistency between the data presented and the findings. |
| 31. | Clarity of major themes | Were major themes clearly presented in the findings? | Yes, major themes were clearly presented in the results section. |
| 32. | Clarity of minor themes | Is there a description of diverse cases or discussion of minor themes? | No, there is not a description of minor themes. |

^a^Tong A, Sainsbury P, Craig J. Consolidated criteria for reporting qualitative research (COREQ): a 32-item checklist for interviews and focus groups. International journal for quality in health care. 2007 Dec 1;19(6):349-57.
