# Supplementary material for: A qualitative evaluation of barriers and facilitators to a large-scale antithrombotic stewardship intervention in the United States Veterans Healthcare system
Source: Int J Clin Pharm. 2025 Jun 4;47(6):1710–9. doi: 10.1007/s11096-025-01922-2 (PMC12630269; doi:10.1007/s11096-025-01922-2)
Supplement: Supplementary file 3 — Supplementary file3 (DOCX 23 kb) [file 11096_2025_1922_MOESM3_ESM.docx]

**Supplementary File 3: Codebook**

| Name | Description |
| --- | --- |
| I. Innovation Domain | Perceptions of the Innovation itself, e.g., a new clinical treatment, educational program, or city service. |
| A. Innovation Source | The group that developed and/or visibly sponsored use of the innovation is reputable, credible, and/or trustable. |
| B. Innovation Evidence-Base | The innovation has robust evidence supporting its effectiveness. |
| C. Innovation Relative Advantage | The innovation is better than other available innovations or current practice. |
| D. Innovation Adaptability | The innovation can be modified, tailored, or refined to fit local context or needs. |
| E. Innovation Trialability | The innovation can be tested or piloted on a small scale and undone. |
| F. Innovation Complexity | The innovation is complicated, which may be reflected by its scope and/or the nature and number of connections and steps. |
| G. Innovation Design | The innovation is well designed and packaged, including how it is assembled, bundled, and presented. |
| H. Innovation Cost | The innovation purchase and operating costs are expensive. |
| II. Outer Setting Domain | Perceptions of the Outer Setting, the setting in which the Inner Setting exists, e.g., hospital system, school district, state. There may be multiple Outer Settings and/or multiple levels within the Outer Setting (e.g., community, system, state). |
| A. Critical Incidents | Large-scale and/or unanticipated events disrupt the Outer Setting during implementation and/or delivery of the innovation. |
| B. Local Attitudes | Sociocultural values (e.g., shared responsibility in helping recipients) and beliefs (e.g., convictions about the worthiness of recipients) encourage the Outer Setting to support implementation and/or delivery of the innovation. |
| C. Local Conditions | Economic, environmental, political, and/or technological conditions enable the Outer Setting to support implementation and/or delivery of the innovation. |
| D. Partnerships & Connections | The Inner Setting is networked with external entities, including referral networks, academic affiliations, and professional organization networks. |
| E. Policies & Laws | Legislation, regulations, professional group guidelines and recommendations, or accreditation standards support implementation and/or delivery of the innovation. |
| F. Financing | Funding from external entities (e.g., grants, reimbursement) is available to implement and/or deliver the innovation. |
| G. External Pressure | Use this to capture themes related to External Pressure that are not included in subconstructs. |
| 1. Societal Pressure | Mass media campaigns, advocacy groups, or social movements or protests drive a need to implement and/or deliver the innovation. |
| 2. Market Pressure | A need to compete with and/or imitate peer entities drives implementation and/or delivery of the innovation. |
| 3. Performance-Measurement Pressure | Quality or benchmarking metrics or established service goals drive implementation and/or delivery of the innovation. |
| III. Inner Setting Domain |  |
| A. Structural Characteristics | Use this to capture themes related to Structural Characteristics that aren't included in subconstructs. |
| 1. Physical Infrastructure | Layout and configuration of space and other tangible material features supports implementation and/or delivery of the innovation. |
| 2. Information Technology Infrastructure | Technological systems for tele-communication, electronic documentation, and data storage, management, reporting, and analysis supports implementation and/or delivery of the innovation. |
| 3. Work Infrastructure | Organization of tasks and responsibilities, within and between individuals and teams, supports implementation and/or delivery of the innovation. |
| B. Relational Connections | Formal and informal relationships, networks, and teams within and across Inner Setting boundaries (e.g., structural, professional) support implementation and/or delivery of the innovation. |
| C. Communications | Formal and informal information sharing practices support implementation and/or delivery of the innovation. |
| D. Culture | Use this to capture themes related to Culture that are not included in subconstructs. |
| 1. Human Equality-Centeredness | There are shared values, beliefs, and norms about the inherent equal worth and value of all human beings. |
| 2. Recipient-Centeredness | There are shared values, beliefs, and norms around caring, supporting, and addressing the needs and welfare of recipients. |
| 3. Deliverer-Centeredness | There are shared values, beliefs, and norms around caring, supporting, and addressing the needs and welfare of deliverers. |
| 4. Learning-Centeredness | There are shared values, beliefs, and norms around psychological safety, continual improvement, and using data to inform practice. |
| E. Tension for Change | The current situation is intolerable and needs to change. |
| F. Compatibility | The innovation fits with workflows, systems, and processes. |
| G. Relative Priority | Implementing and delivering the innovation is important compared to other initiatives. |
| H. Incentive Systems | Tangible and/or intangible incentives and rewards and/or disincentives and punishments support implementation and delivery of the innovation. |
| I. Mission Alignment | Implementing and delivering the innovation is in line with the overarching commitment, purpose, or goals of the Inner Setting. |
| J. Available Resources | Use this to capture themes related to Available Resources that are not included in subconstructs. |
| 1. Funding | Funding is available to implement and deliver the innovation. |
| 2. Space | Physical space is available to implement and deliver the innovation. |
| 3. Materials & Equipment | Supplies are available to implement and deliver the innovation. |
| K. Access to Knowledge & Information | Guidance and/or training is accessible to implement and deliver the innovation. |
| IV. Individuals Domain | Perceptions about Individuals, including their roles and characteristics. Note: These may be self-perceptions for some roles. |
| A. High-Level Leaders | Individuals with a high level of authority, including key decision-makers, executive leaders, or directors. |
| B. Mid-level Leaders | Individuals with a moderate level of authority, including leaders supervised by a high-level leader who supervise others. |
| C. Opinion Leaders | Individuals with informal influence on the attitudes and behaviors of others. |
| D. Implementation Facilitators | Individuals with subject matter expertise who assist, coach, or support implementation. |
| E. Implementation Leads | Individuals who lead efforts to implement the innovation. |
| F. Implementation Team Members | Individuals who collaborate with and support the Implementation Leads to implement the innovation, ideally including Innovation Deliverers and Recipients. |
| G. Other Implementation Support | Individuals who support the Implementation Leads and/or Implementation Team Members to implement the innovation. |
| H. Innovation Deliverers | Individuals who are directly or indirectly involved with delivering the innovation. Potential deliverers |
| I. Innovation Recipients | Individuals who are directly or indirectly receiving the innovation. |
| J. Need | The individual(s) has deficits related to survival, well-being, or personal fulfillment, which will be addressed by implementation and/or delivery of the innovation. |
| K. Capability | The individual(s) has interpersonal competence, knowledge, and skills to fulfill Role. |
| L. Opportunity | The individual(s) has availability, scope, and power to fulfill Role. |
| M. Motivation | The individual(s) is committed to fulfilling Role. |
| V. Implementation Process | Perceptions of the Implementation Process, i.e., the activities and strategies used to implement the innovation. |
| A. Teaming | Join together, intentionally coordinating and collaborating on interdependent tasks, to implement the innovation. |
| B. Assessing Needs | Use this to capture themes related to Assessing Needs that are not included in subconstructs. |
| 1. Innovation Deliverers | Collect information about the priorities, preferences, and needs of deliverers to guide implementation and delivery of the innovation. |
| 2. Innovation Recipients | Collect information about the priorities, preferences, and needs of recipients to guide implementation and delivery of the innovation. |
| C. Assessing Context | Collect information to identify and appraise barriers and facilitators to implementation and delivery of the innovation. |
| D. Planning | Identify roles and responsibilities, outline specific steps and milestones, and define goals and measures for implementation success in advance. |
| E. Tailoring Strategies | Choose and operationalize implementation strategies to address barriers, leverage facilitators, and fit context. |
| F. Engaging | Use this to capture themes related to Engaging that are not included in subconstructs. |
| 1. Innovation Deliverers | Attract and encourage deliverers to serve on the implementation team and/or to deliver the innovation. |
| 2. Innovation Recipients | Attract and encourage recipients to serve on the implementation team and/or participate in the innovation. |
| G. Doing | Implement in small steps, tests, or cycles of change to trial and cumulatively optimize delivery of the innovation. |
| H. Reflecting & Evaluating | Use this to capture themes related to Reflecting & Evaluating that are not included in subconstructs. |
| 1. Implementation | Collect and discuss quantitative and qualitive information that indicates the degree to which implementation outcomes are achieved. |
| 2. Innovation | Collect and discuss quantitative and qualitative information that indicates the degree to which innovation outcomes are achieved. |
| I. Adapting | Modify the innovation and/or the Inner Setting for optimal fit and integration into delivery routines. |
| V. Additional Codes |  |
| A. Burnout | More emotional mention of not having enough time, feeling overwhelmed |
| B. Fidelity | Fidelity to the innovation (i.e., VIONE) |
| C. Awareness of Innovation | How familiar interviewee is with innovation |
| D. Voluntold | Being told vs. being asked/volunteering to particpate in the implementation and/or innovation |
| E. Initiative Elements |  |
| 1. Consult-PADR | Revised templated text for anticoagulation consults and/or prior authorization drug review (PADR) requests |
| 2. Note Template-View Alert | Revised templated text for in/outpatient anticoagulation note templates/view alerts that addressed DOAC-antiplatelet use |
| 3. In-Service | Informed other services/provided an in-service about initiative |
| 4. Champion | Identified champion and/or partnered with another service to support the initiative (what was their role?) |
| 5. Flag | DOAC population management tool/DOAC dashboard DOAC-antiplatelet flag incorporated into processes |
| 6. E-Consult | Created an e-Consult to Cardiology or other service (specify) to address combination therapy |
| 7. Clinical Reminder | Clinical reminder |
| 8. Letter | Provider and patient letter examples for Care in the Community or non-VA provider (self-directed care) |
| 9. Other Initiative Element | Anything else done to support the initiative not represented in the elements above. |

**Article information:**

Barriers and facilitators associated with a large-scale antithrombotic stewardship intervention to improve appropriate use of combination anticoagulation-antiplatelet therapy: a qualitative study

*International Journal of Clinical Pharmacy*

Jacob E. Kurlander^1, 2^; Claire H. Robinson^1^; David Parra^3^; Lacey Evans^1^; Von Moore^4^; Geoffrey D. Barnes^2^; Allison A. Ranusch^1^; Jeremy B. Sussman^1,2^

1. VA Ann Arbor Healthcare System Center for Clinical Management Research
2. Department of Internal Medicine, University of Michigan, Ann Arbor, MI, USA
3. Department of Veterans Affairs, Veterans Integrated Service Network 8, Pharmacy Benefits Management, Tampa, FL
4. VA Center for Medication Safety, Pharmacy Benefits Management Services, Hines, Illinois

**Corresponding Author:**

Jacob Kurlander, MD, MS

VA CCMR, VA Ann Arbor Healthcare System (152)

PO Box 130170

Ann Arbor, MI 48113-0170

jkurland@med.umich.edu
